# Exons deletion of *CNKSR2* gene identified in X-linked syndromic intellectual disability

**DOI:** 10.1186/s12881-020-01004-2

**Published:** 2020-04-03

**Authors:** Mei Daoqi, Chen Guohong, Wang Yuan, Yang Zhixiao, Xu Kaili, Mei Shiyue

**Affiliations:** 1 Department of of Eastern Neurology, Children’s Hospital Affiliated to Zhengzhou University, Zhengzhou Children’s Hospital, Zhengzhou, China; 2 Department of Intensive Care Unit, Henan Provincial Key Laboratory of Children’s Genetics and Metabolic Diseases, Children’s Hospital Affiliated to Zhengzhou University, Zhengzhou Children’s Hospital, Zhengzhou, China

**Keywords:** X-linked syndromic mental retardation, Intractable seizures, *CNKSR2* gene, Exons deletion

## Abstract

**Background:**

The Houge type of X-linked syndromic mental retardation is an X-linked intellectual disability (XLID) recently recorded in the Online Mendelian Inheritance in Man (OMIM) and only 8 cases have been reported in literature thus far.

**Case presentation:**

We present two brothers with intractable seizures and syndromic intellectual disability with symptoms consisting of delayed development, intellectual disability, and speech and language delay. The mother was a symptomatic carrier with milder clinical phenotype. Whole exome sequencing identified a small fragment deletion spanning four exons, about 9.5 kilobases (kb) in length in the *CNKSR2* gene in the patients. The mutation co-segregation revealed that exon deletions occurred de novo in the proband’s mother.

**Conclusion:**

Although large deletions have been reported, no small deletions have yet been identified. In this case report, we identified a small deletion in the *CNKSR2* gene. This study enhances our knowledge of the *CNKSR2* gene mutation spectrum and provides further information about the phenotypic characteristics of X-linked syndromic intellectual disability.

## Background

X-linked intellectual disability (XLID), also known as X-linked mental retardation (XLMR), refers to a generalized neurodevelopmental disorder caused by a gene defect on the X chromosome. XLID accounts for over 10% of the cases in males with intellectual disability [1]. It is a genetically and clinically heterogeneous disorder. With extensive genetic studies, more than 100 genes have been proposed to be associated with XLID [2, 3]. Depending on whether additional abnormalities are present, XLID can be roughly subdivided into syndromic and non-syndromic forms. The Houge type of X-linked syndromic mental retardation (MRXSHG) is an XLID that was recently recorded in OMIM (#301008) in 2017. MRXSHG is caused by a deficiency of the *CNKSR2* gene and only 8 cases have been reported in literature. This case report discusses a Chinese family of which two brothers were characterized with intractable seizures, developmental delay, and speech and language delay. In addition, the mother was a symptomatic carrier with milder phenotype. A small deletion of around 9.5 kilobases (kb) in length in the *CNKSR2* gene was identified using whole exome sequencing (WES).

## Case presentation

**Fig. 1:**
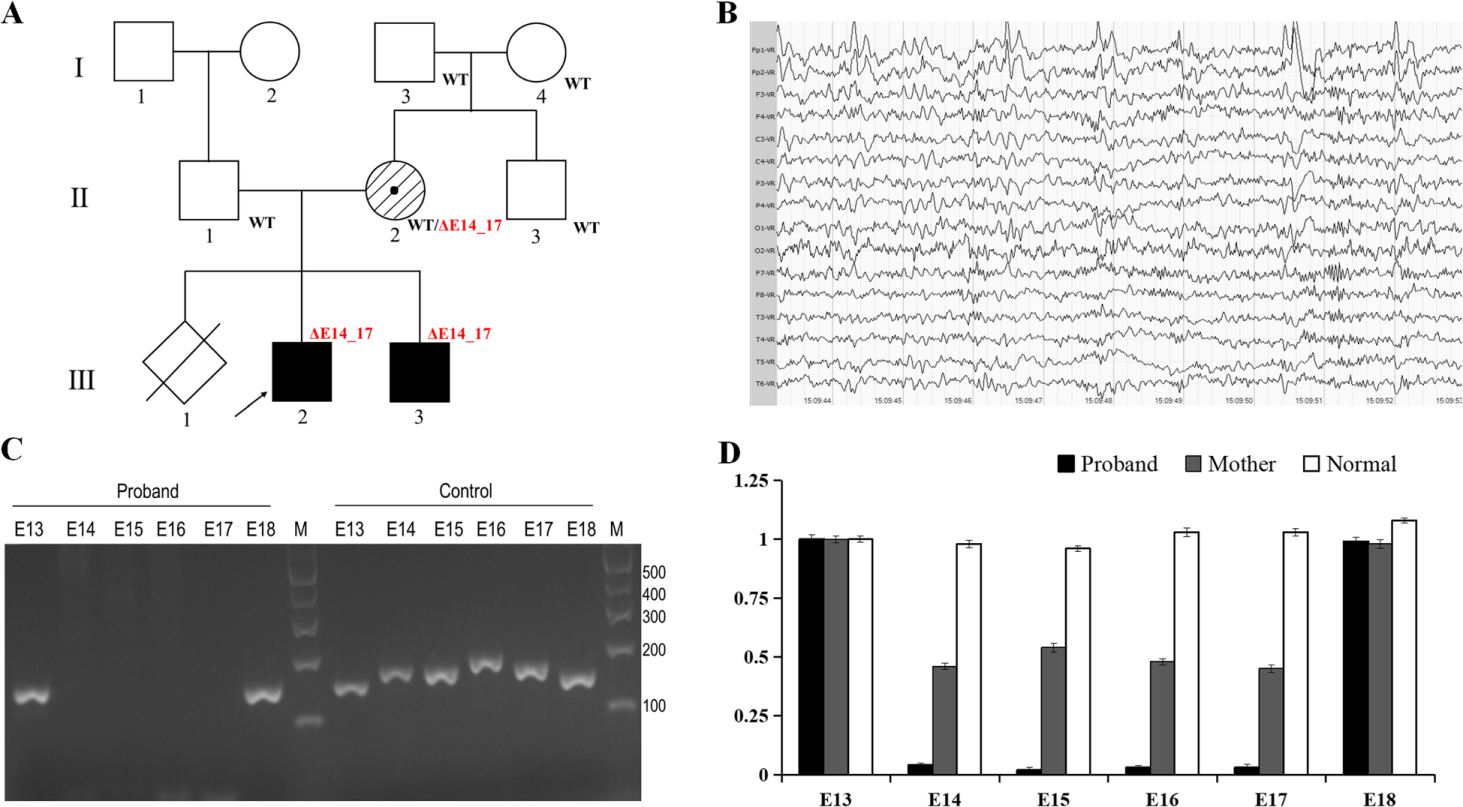
**a** The mutation co-segregation in the pedigree. **b** Sleep–electroencephalography (EEG) showed that low-middle amplitude sharp waves and slow spike waves were distributed sporadically or continuously in the frontal and lateral temporal cortices during the interictal phase. **c** Exon 14–17 deletion in the proband was verified by PCR and agarose gel electrophoresis. PCR production for the exon 14–17 could not be detected in proband, but the adjacent exon 13 and 18 could be amplified. While the healthy control child gave products with expected sizes. **d** Exon 14–17 deletion was hemizygous in the proband, and heterozygous in the mother validated by quantitative realtime PCR

The proband (III2 in Fig. 1a), G3P1, was a 5.5-year-old boy of Han Chinese descent. He was admitted to the Neurology Department with the complaint of medically intractable seizures and developmental delay. History of his subsisting illness included generalized seizures, attention deficit hyperactivity disorder and weakness in lower extremities. The parents denied any history of infectious diseases or consumption of contaminated water in the past few years. Central nervous system infection was also excluded by routine laboratory tests, which included measurements of complete blood count, serum electrolytes, calcium, procalcitonin, C-reactive protein, and glucose. According to past medical history, the proband was full-term and naturally delivered in hospital with no abnormal indices observed after birth. At 11 months old, he was found to have low muscular tension. He began taking his first steps at 18 months old and was walking and sitting independently until he was 25 months old, with weakness on both sides of his limbs. The proband’s first words were spoken at 26 months old and he was only able to speak a few single words even at 3 years old. From these findings, it was evident that his psychomotor and language development was delayed. The seizure onset occurred at 4 years old, characterized by uncontrollable jerking of the limbs and face, followed by a tonic upward gaze, cyanosis of the lips and face, and loss of consciousness. The epilepsy could subside naturally without intervention and the average duration of each episode was 1 to 2 min with occurrences at intervals of 2–3 weeks. At 4.5 years old, he was unable to properly control his bladder or bowels and occasionally had problems with his balance, evident by unstable walking. During that period, he was treated with rehabilitation therapy for approximately 6 months in a local traditional Chinese medical clinic. The proband’s motor and language skills were improving with treatment according to the father, but the composition of the drugs administered was not clear.

In our Neurology Department, the proband was assessed by the Denver Developmental Screening Test (DDST) to evaluate his developmental progress. Five separate domains including Gross-motor, Fine-motor, Adaptive, Language and Personal-social were evaluated. The proband was delayed in all the development domains with an average Developmental Quotient (DQ) score of 29, much lower than healthy counterparts of the same age. Electromyography showed that nerve conduction velocity in the lower extremity was slow. Magnetic resonance imaging (MRI) of the head and neck showed high signal intensity on T1-weighted images and low signal intensity on T2-weighted images in the bilateral corpus callosum (data is not shown). These findings indicated that the proband had white matter lesions. To record brain electrical activity and physical behaviors during seizures, long term video-Electroencephalography (EEG) monitoring was utilized. The background EEG activities had a slow spike-and-wave and characterized by a bisynchronous, symmetric discharge with a frequency of 6–7 Hz, mixed with a few fast spike-waves. There was no obvious changes in the slow spike-and-wave during hyperventilation and intermittent photic stimulation. During the interictal phase, as shown in Fig. 1b, a lot of low-middle amplitude sharp waves and slow spike waves were distributed sporadically or continuously in the frontal and lateral temporal cortices. Consequently, he was diagnosed with a generalized seizure. Valproate sodium was orally administered with a dose of 30 mg/kg/d at 12 h intervals. Follow-up reports showed that no seizure occurred in the past 10 months.

His younger brother (III3) showed mild intellectual disability and attention deficit hyperactivity disorder. He could walk without assistance and learn independently. Denver Developmental Screening Test also showed milder developmental delay. He began experiencing seizures at 3-years-old, but the seizures did not occur as frequently and severely as the proband. Brain MRI showed small multifocal white matter lesions. In general, the younger brother showed less severe symptoms and was never treated with anti-seizure drugs. For the proband’s mother (II2), her symptoms were much milder. Development of her intelligence was slightly delayed, with poor language expression, slowed speech response and poor social communication skills. Starting from junior high school, her limbs would occasionally shake uncontrollaby and she would wake up suddenly during her sleep. The frequency of these episodes accelerated from twice a year to once a month. Other developmental conditions of the mother were found to be acceptable and bearable. She was not treated with anti-seizure drugs at the time due to poor financial conditions. Her first pregnancy ended in a spontaneous miscarriage at 15 weeks’ gestation due to unknown causes. No one else in the pedigree was found with any history of seizures or developmental delay.

To explore genetic factors contributing to the intellectual disability and seizures of the proband, whole exome sequencing of the proband and parents (Trio-WES) was performed by a commercial company (Chigene Inc., Beijing, China). In brief, genomic DNA was extracted from peripheral blood lymphocytes and used as template for library construction. The whole exomes were enriched by a biotinylated capture probe. Subsequently, the pre-captured libraries were pooled together, quantified and then sequenced on Illumina Hiseq 2500 sequencer.

Through Trio-WES, no definite or suspected pathogenic substitutions, small insertions or deletions associated with clinical symptoms were detected. For further analysis, we found that there were no reads mapped to exon 14_17 of the *CNKSR2* gene (RefSeq: NM_014927) in the proband. It suggested that there was a deletion of exon 14_17. Exons deletion in the X-linked *CNKSR2* in the proband was verified by PCR with gel electrophoresis. Primer sequences and PCR conditions are available on request. As expected, PCR production for the exons 14, 15, 16 and 17 could not be detected in the proband and his brother, but the adjacent exons 13 and 18 could be amplified (Fig. 1c). PCR for each exon from 13 to 18 in proband’s father and healthy control children yielded products with expected sizes. PCR products were sequenced and aligned to the reference sequence. Walking Primer PCR was used to define the breakpoints and 9.5 kb of deletion was detected. A pedigree genetic test showed that the mother harbored heterozygous exon deletions validated by quantitative real-time PCR (Fig. 1d) and that the maternal grandparents and uncle did not carry the exon deletions. It indicated that the exon 14_17 deletion occurred de novo in the proband’s mother. The mother is a symptomatic carrier with a milder clinical phenotype. The mutation co-segregation is consistent to the X-linked hereditary mode in genetics. To our knowledge, exon 14_17 deletion is a novel mutation and no cases have been reported in the literature thus far.

## Discussion and conclusions

Numerous researchers have focused on the genetics of XLID where more than 100 genes have been reported [2–4]. The Houge type of X-linked syndromic mental retardation (MRXSHG; #301008), firstly identified by Houge in 2012 [5], is a XLID recently recorded in OMIM. It is characterized by delayed development, intellectual disability, speech and language delay, and early-onset seizures. Frequency of the *CNKSR2* mutation was reported to occur in 1 of 112 patients with X-linked epilepsy-aphasia spectrum [6]. MRXSHG is caused by deficiency of the *CNKSR2* gene located in Xp22.12. The *CNKSR2* gene, encoding 1034 amino acids, is only highly expressed in brain and acts as a scaffold and adaptor protein in the neuronal postsynaptic density [7, 8]. *CNKSR2* was proposed to inhibit Ras/MAPK signaling [9], hence the name “*CNKSR2*” (Connector Enhancer Of Kinase Suppressor Of Ras 2). *CNKSR2* was also speculated to be connected with N-methyl-d-aspartate receptors and a factor towards Epilepsy-aphasia spectrum [10, 11]. Since the first case reported by Houge et al., only 8 mutations consisting of 5 gross deletions from 0.234 Mb to 1.17 Mb in length, 1 small insertion (D152RfsX8) and 2 nonsense mutations (Arg712Ter and Arg729Ter) have been identified by Chromosomal microarray analysis (CMA), X-chromosome exome sequencing or PCR-Sanger sequencing, respectively (Table 1) [5–7, 10, 12].

**Table 1:** Genetic and clinical details of 9 families with CNKSR2 deficiency

Case ID	CNKSR2 mutation	Number of patients	Affected patients	Developmental delay	Speech delay	Intellectual disability	Epilepsy/seizures	ADHD	Brain MRI	Sleep-EEG	Reference
Famlily 1	1.17 Mb deletion (20,297,696–21,471,387)	3	proband	yes	yes	yes	yes	yes	normal	CSWS	[7]
			brother	yes	yes	yes	yes	yes	normal	CSWS	
			mother	no	no	mild	no	no	normal	normal	
Famlily 2	0.51 Mb deletion (21,193,947–21,707,169)	2	proband	yes	yes	yes	yes	yes	no specific	normal	[7]
			brother	yes	yes	yes	no	yes	normal	normal	
Famlily 3	0.342 Mb deletion (21,328,677–21,670,497)	1	proband	yes	yes	yes	yes	N/A	normal	CSWS	[12]
Famlily 4	0.234 Mb deletion (21,375,312–21,609,484)	1	proband		yes	yes	yes	yes	normal	CSWS	[7]
Famlily 5	0.234 Mb deletion (21,285,233–21,519,405)	1	proband	yes	yes	yes	yes	yes	normal	N/A	[5]
Famlily 6	frameshift mutation (g.21,458,832_3insA, p.D152RfsX8)	3	proband	yes	yes	yes	yes	yes	ND	ND	[7]
			brother	yes	mild	yes	Febrile	yes	minor cortical atrophy	N/A	
			brother	yes	yes	yes	yes	yes	N/A	N/A	
Famlily 7	nonsense mutation (c.2185C>T, p.Arg729Ter)	1	proband	yes	yes	yes	yes	yes	normal	CSWS	[10]
Famlily 8	nonsense mutation (c.2314 C>T;p.Arg712Ter)	5	proband	yes	yes	yes	yes	yes	normal	CSWS	[6]
			brother	N/A	mild	mild	yes	yes	normal	CSWS	
			sister	N/A	mild	mild	mild	no	N/A	N/A	
			mother	no	no	no	febrile	no	N/A	N/A	
			maternal uncle	yes	yes	mild-to-moderate	yes	yes	N/A	N/A	
Present Famlily	9.5 Kb deletion (21606698-21616207)	3	proband	yes	yes	yes	yes	yes	white matter lesions	CSWS	This case
			brother	yes	yes	yes	yes	yes	small multifocal white matter lesions	CSWS	
			mother	no	mild	mild	Febrile	no	normal	ND	

*ADHD* attention deficit and hyperactivity disorder: *N/A* not available, *ND* not done, *CSWS* continuous spike-and-slow-waves

Presently, CMA is recommended as the first-tier clinical diagnostic test for individuals with unexplained developmental delay/intellectual disability by American College of Medical Genetics (ACMG) [13]. CMA can sensitively detect copy number variants (CNVs) with a high-resolution of 100 ∼ 400 kb, depending on the coverage of CMA probes. The development of next-generation sequencing, WES, has greatly improved the diagnostic yield in rare pediatric diseases. WES is an effective strategy to identify genetic mutations, such as single nucleotide variants and small insertions/deletions. In addition, CNV sequencing has been introduced to WES, although there can be a high level of bias and noise. In our diagnostic test, we chose to to conduct WES and detected 4 exon deletions at around 9.5 kb in length. If the CMA was chosen, the small deletion may have been omitted due to an insufficient amount of CMA probes covered.

In a previous study, the MRXSHG patients were all males whereas the female mutation carriers were unaffected, aside from two symptomatic carriers reported by Damiano et al. [6]. According to Lyon’s hypothesis [14], females have two X-chromosomes where only one is packaged and transcriptionally inactivated in a random way. X-inactivation pattern analysis showed a significant skewing of X-inactivation ratios (85%:15%)in peripheral blood cells of the mother, using Hpa II predigestion of DNA followed by PCR of the highly polymorphic CAG repeat of the *AR* gene. This skewed X-inactivation ratio may explain the phenotypes of female symptomatic carriers. In this family, the correlation between phenotype and genotype was consistent with the X-linked transmission pattern of MRXSHG summarized by Damiano et al. [6].

In the present study, we described the clinical features and genetic analysis of a family in which the two male patients were severely affected with intractable seizures and syndromic mental retardation, and their mother was a symptomatic carrier with focal seizure with mild language and intellectual impairment. Through Trio-WES, a small fragment deletion spanning four exons, about 9.5 kb in length, was detected in the patients. The deletion was predicted to lead a loss of function. Although large deletions have been reported, no small deletions have yet been identified in literature. This study discovered a small deletion in MRXSHG, which adds to the mutation spectrum of *CNKSR2* and improves our understanding of the phenotypic characteristics of X-linked syndromic intellectual disability.

## Data Availability

The data that support the findings of this study are available from the authors upon reasonable request and with permission of The Review Board of Zhengzhou Children’s Hospital.

## Abbreviations

XLID: X- linked intellectual disability
XLMR: X-linked mental retardation
MRXSHG: The Houge type of X-linked syndromic mental retardation
CNKSR2: Connector Enhancer Of Kinase Suppressor Of Ras 2
DDST: Denver Developmental Screening Test
MRI: Magnetic resonance imaging
EEG: Electroencephalography
DQ: Developmental QuotientCMA: Chromosomal microarray analysis
WES: whole exome sequencing
Kb: kilobases
CNV: copy number variants
ACMG: American College of Medical Genetics
